# Exploratory evaluation of an eye-tracking system in patients with advanced spinal muscular atrophy type I receiving nusinersen

**DOI:** 10.3389/fneur.2022.918255

**Published:** 2022-09-30

**Authors:** Yukako Yae, Kotaro Yuge, Toshiyuki Maeda, Fumio Ichinose, Muneaki Matsuo, Osamu Kobayashi, Kazuo Okanari, Yusei Baba, Chihiro Yonee, Shinsuke Maruyama, Minoru Shibata, Tatsuya Fujii, Madoka Chinen, Yushiro Yamashita

**Affiliations:** ^1^Department of Pediatrics and Child Health, Kurume University School of Medicine, Kurume, Fukuoka, Japan; ^2^Department of Pediatrics, Faculty of Medicine, Saga University, Saga, Japan; ^3^Department of Pediatrics, Oita University Faculty of Medicine, Yufu, Oita, Japan; ^4^Department of Pediatrics, Kagoshima University Graduate School of Medical and Dental Sciences, Kagoshima, Japan; ^5^Department of Pediatrics, Shiga Medical Center for Children, Moriyama, Japan; ^6^Biogen Japan Ltd., Tokyo, Japan

**Keywords:** eye-tracking, nusinersen, spinal muscular atrophy, advanced, evaluation method, observational study

## Abstract

**Objective:**

This study evaluated the feasibility of a matching-pair test using eye-tracking technology to assess nusinersen effectiveness in patients with advanced spinal muscular atrophy (SMA) type I.

**Methods:**

This prospective, observational study enrolled patients with 5q-SMA type I who had lost gross motor function. Three different levels of matching-pair tests were conducted using the eye-gaze system (My Tobii; TobiiDynavox Inc.) at baseline, and after 9 and 24 weeks of nusinersen treatment. The primary endpoint was the change from baseline in matching-pair test scores and response times (i.e., the time to answer matching-pair test) at 24 weeks from baseline. Children's Hospital of Philadelphia Infant Test of Neuromuscular Disorders (CHOP-INTEND), Pediatric Quality of Life inventory for patients with Neuromuscular Disease (PedsQL-NM) and Numerical Rating Scale (NRS) scores were also assessed as secondary endpoints. Analysis of ocular fixation was performed as an additional analysis. This study was registered at https://www.umin.ac.jp/ctr/ (UMIN000033935).

**Results:**

Seven patients (one male, six female) aged 5–21 years (median 11 years) were enrolled; all patients were bedridden and six patients were ventilated. All seven patients were able to conduct level 1 matching-pair tests at each assessment; five patients were also able to conduct levels 2 and 3. Two patients (those with the highest CHOP-INTEND scores) were able to complete all tests correctly within 60 s. There was a non-significant trend toward improvement in CHOP-INTEND, PedsQL-NM, and NRS scores over the 6-month period. There were no significant differences in the number of actions, errors, correct answers, or response times between baseline and Week 9 or 24 at any level. However, the result of an additional analysis suggests that detection of eye movement would be useful to evaluate for advanced SMA.

**Conclusions:**

Eye-tracking systems are possibly feasible for the assessment of treatment efficacy in patients with advanced SMA type I.

## Introduction

Spinal muscular atrophy (SMA) is an autosomal recessive neuromuscular disorder that causes degeneration of motor neurons, leading to progressive muscle atrophy and weakness (1). In approximately 95% of patients, SMA is caused by a homozygous deletion in exon 7 of the survival motor neuron 1 (*SMN1*) gene located on chromosome 5q13, which results in reduction of SMN protein expression, and degeneration of motor neurons of the spinal cord (1).

SMA is classified into four severity grades (I–IV) based on the age of onset and achieved motor function (2, 3). Approximately 50% of patients with SMA have type I disease (the most severe type) and present with hypotonia and loss of tendon reflexes, poor head control, and predominantly proximal symmetrical flaccid quadriparesis preferentially affecting lower limbs (4, 5). Patients with SMA type I develop chronic respiratory failure and bulbar dysfunction, and the median time to the composite outcome of death or mechanical ventilation for ≥16 h/day is 7.7 months (6).

Three SMN-dependent therapies have been approved in Japan—nusinersen, risdiplam, and onasemnogene abeparvovec. These therapies have dramatically changed the lives of SMA patients, particularly those with early-onset SMA type I. For instance, the pivotal studies for nusinersen (ENDEAR) and risdiplam (FIREFISH) showed marked improvements in motor function in infants with SMA who started treatment before the age of 7 months (7, 8). Of note, these pivotal clinical trials excluded infants with impaired pulmonary function, such as those receiving invasive ventilation or tracheostomy; however, many patients with advanced SMA type I are bedridden and mechanically ventilated in real-world settings (9). Tracheostomy or mechanical ventilation in severe SMA type I patients are not recommended in several countries due to their impact on patient quality of life (QoL), risk of complications, ethical questions around prolonging life when there is no likelihood of improvement, or religious reasons (6, 10–12). A survey in the United States found that 29.5% of patients with SMA type I have a tracheostomy (13), while in Japan, 97.9% have tracheostomy and physicians tend to choose life-sustaining care (14, 15), so the number of patients with advanced SMA type I is expected to be higher in Japan than in other countries.

Although measurement scales for gross motor functions, have been used in clinical trials for the treatment of SMA, these are not suitable for evaluating treatment effects in advanced patients who have lost almost all gross motor function. Therefore, it is important to develop measurement scales of fine motor function to assess the effectiveness of treatment and its impact on QoL in patients with advanced SMA type I (16). It has been reported that normal oculomotor function is preserved in motor neuron disease (MND) patients as well as SMA patients (17–19), so eye-tracking systems are used as communication tools in patients with amyotrophic lateral sclerosis (ALS) (20, 21), locked-in syndrome (22), and SMA (16, 19). In SMA, it is reported that eye movement is preserved in types II and III (17), but some individual cases, reported before the availability of genetic testing, have suggested extraocular muscle dysfunction in SMA (23–26). Further, it is also reported that ALS patients show oculomotor dysfunction (27, 28), which may be useful as a biomarker (29).

Pair-matching tasks using an eye tracker device have previously been used to evaluate cognitive performance in patients with SMA type I (30), and we hypothesized that such tests could also be useful for the measurement of treatment effects in patients with advanced SMA type I. Therefore, we conducted a pilot study to evaluate whether pair-matching tasks using an eye-tracker device are useful to assess treatment effectiveness.

## Materials and methods

### Study design and participants

This prospective, multicenter, observational study enrolled patients with SMA type I who were scheduled to start nusinersen treatment. Patients and/or parents received information about the study and parents (or the patient's legally authorized representative) provided written informed consent prior to participation in the study. The study was approved by the institutional review boards of all participating hospitals, as well as the Ethics Committee of the Kurume University (reference 18103). The study was registered at the University Hospital Medical Information Network Center Clinical Trials Registry (UMIN000033935).

Eligible patients were aged >3 years (considered to be the minimum age to allow patients to understand the task and follow instructions), had genetically confirmed 5q-SMA type I, an Hammersmith Functional Motor Scale Expanded (HFMSE) score of 0 (to ensure inclusion of patients with limited gross motor function), and a plan to start nusinersen treatment. Patients who were unable to perform a matching-pair test, had a history of hypoxic brain injury/epileptic episode, or had respiratory tract infection were excluded.

### Treatment

**Figure 1:**
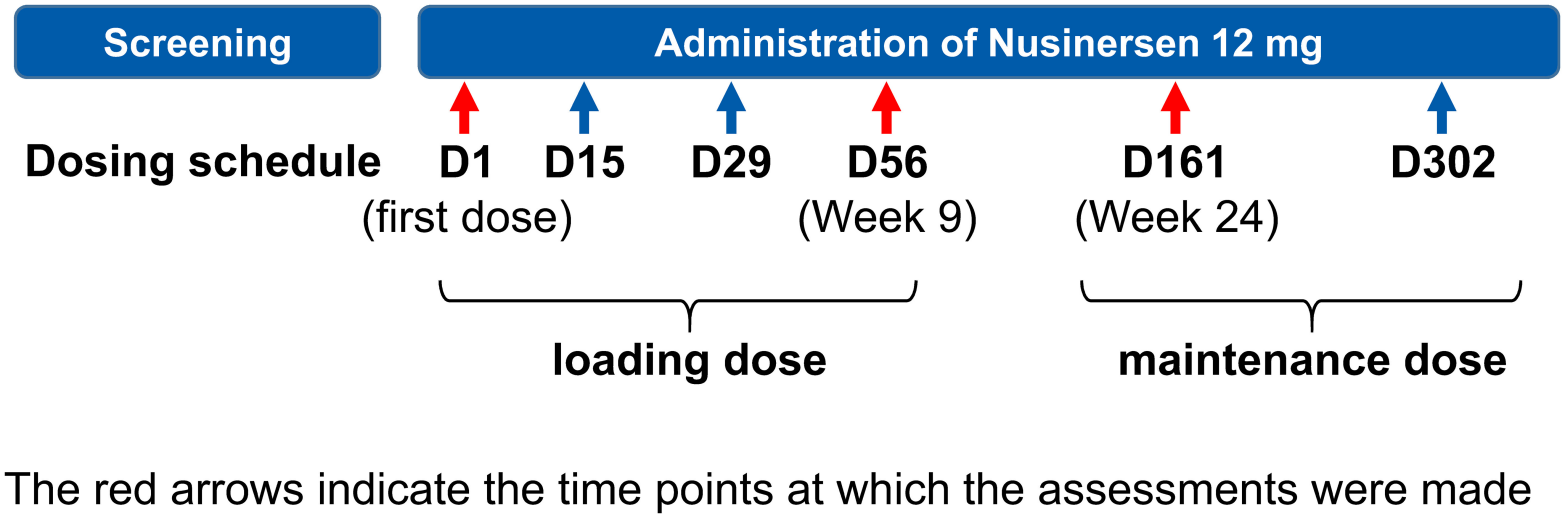
Study design.

Nusinersen was administered intrathecally according to the approved dosage schedule in Japan, i.e., a loading dose of 12 mg on Day 0, and at Weeks 2, 4, and 9, followed by maintenance therapy with 12 mg every 4 months thereafter (Figure 1).

### Study procedures

Study assessments were conducted on Day 0 ± 7 (baseline), Day 56 ± 14 (Week 9–at the end of the loading dose period), and Day 161 ± 14 (Week 24–after the first maintenance dose) of nusinersen treatment. On each assessment day, patients underwent the matching-pair test, and were assessed using Children's Hospital of Philadelphia Infant Test of Neuromuscular Disorders (CHOP-INTEND) (31, 32), Pediatric Quality of Life inventory for patients with Neuromuscular Disease (PedsQL-NM) (33), and a Numerical Rating Scale (NRS). The PedsQL-NM includes 25 items in three core domains: (1) child's disease (17 items related to the disease process and associated symptomatology); (2) communication (three items related to the patient's ability to communicate with health care providers and others about his/her illness); and (3) family resources (five items related to family financial and social support systems) (33). Parents scored each item on a 5-point Likert scale from 0 (never a problem) to 4 (almost always a problem), followed by reverse scoring and linear transformation to a 0–100 scale (i.e., 0=100, 1=75, 2=50, 3=25, 4=0), with an increase in score indicating improvement. Caregivers also rated the severity of 15 systemic symptoms (related to gross motor function, fine motor function, respiration, swallowing, feeding, intestinal motility, sleep, fatigue) on a NRS from 1 to 10, where 1 = extremely severe and 10 = extremely mild; an increase in NRS score indicated clinical improvement. Adverse events (AEs) and serious AEs were also monitored.

### Matching pair-test

#### Eye gaze tracking system

The matching-pair test was conducted using a far-red light-based gaze detector (My Tobii; TobiiDynavox Inc.; commercially available as a communication tool), which was attached under the screen of a laptop (display size 15.6 inch). Once the system is correctly positioned in front of the patient, the Tobii detector tracks the movements of one or both eyes. Image processing software analyzes the Tobii's image of the eye and determines where on the screen the user is looking, based on the relative position of the center of the pupil and the corneal reflection within the Tobii's image.

#### Test set-up and calibration

**Figure 2:**
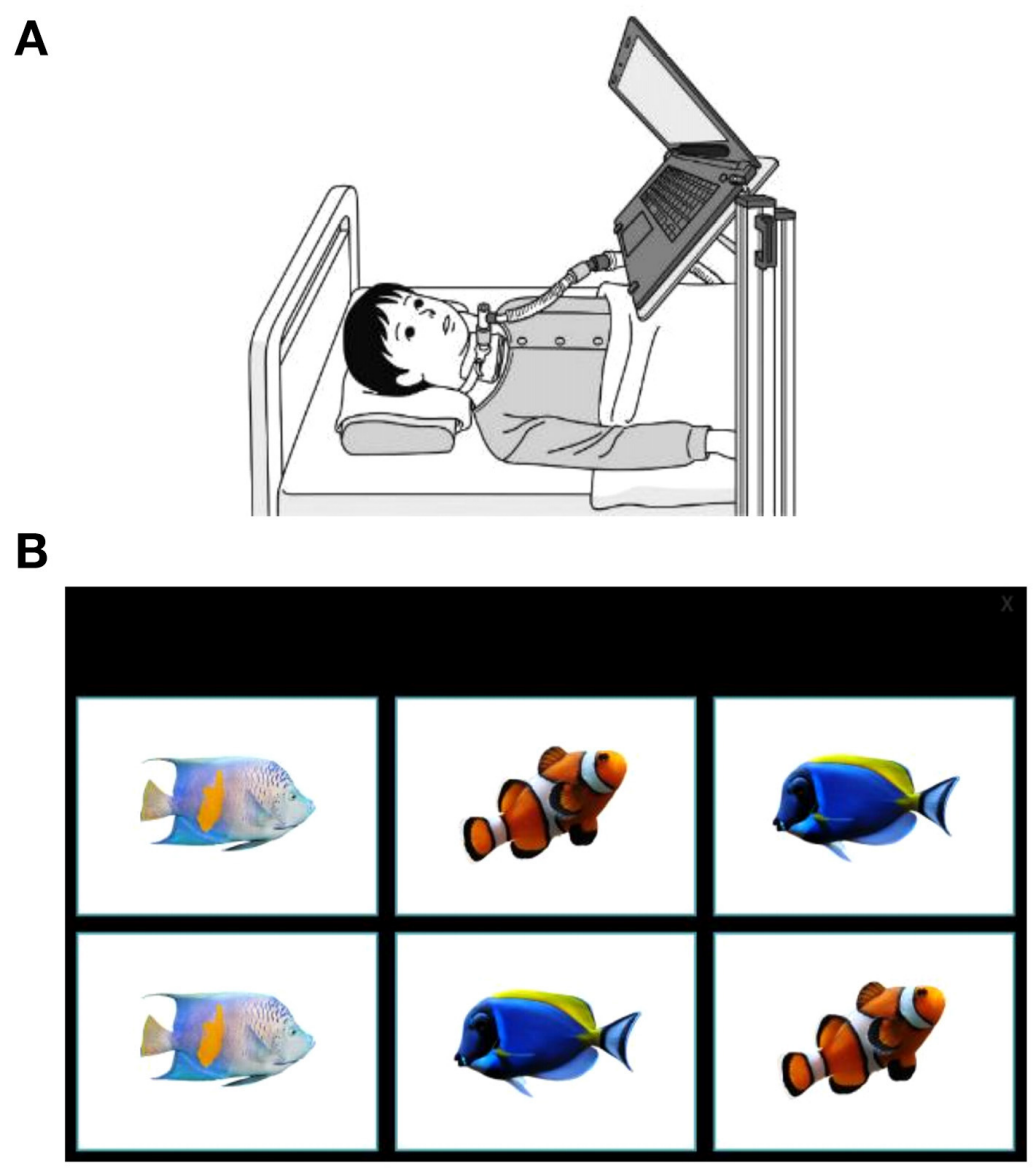
The eye-tracking system and example display of a matching pair-test. **(A)** A laptop was positioned in the patient's eyeline. **(B)** An example of the level 1 matching-pair test in which patients were asked to gaze for 1 s at each image, consecutively identifying matching pairs. The eye-tracking device at the base of the laptop screen captured data on eye movements.

Whenever possible, each matching-pair test was conducted by the same evaluator and at the same time of day, and the test videotaped to be evaluated later. The physician selected appropriate timing for the test, based on the patient's general condition. A laptop computer was fixed to a stand, the height and angle of which was adjusted according to the eye level and posture of each patient so that the same distance and angle were maintained between the screen and the face (Figure 2A). The test was conducted each time at the same fixed setting position.

The patient's pupil position was calibrated after the patient had received an explanation of the test and played a mini-game using the device in order to familiarize them with the device prior to starting the test. Calibration was performed using at least two points to project the gaze accurately and identify each patient's dominant eye using calibration software included in the system. Once calibrated, the patient played one level 1 matching-pair test as a practice.

#### Matching-pair test

The test included three levels (levels 1–3); all patients were asked to start at level 1 and principal investigators determined whether patients could attempt additional levels, depending on the patient's ability.

In level 1, patients were presented with 6 panels (each a square of 10.8 × 7.6 cm) and required to identify 3 sets of matching pairs (Figure 2B); in level 2, patients were given 12 panels (each a square of 6.0 × 4.5 cm) to identify 6 sets of matching pairs; and in level 3, patients had 20 panels (each a square of 4.7 × 3.5 cm) to identify 10 sets of matching pairs. The patient was asked to select a panel by gazing at it for 1 s. Each patient performed the test twice at each level. Because patients with SMA commonly experience fatigue, more tests may cause increased tiredness; therefore, we decided to perform the matching-pair test only twice. The first test allowed the patient to practice performing the tasks correctly but may have made them nervous, so only data from the second test were recorded. If the second test could not be performed, data from the first test were used in the analysis.

The number of errors and correct answers were measured, as well as the time to complete each level. The target time for level completion was 60 s, but if the test was not completed within 60 s, it could be continued until all correct answers had been obtained. If the test was interrupted, the time and the results up to the interruption were recorded. The action number was defined as the number (sum) of correct and incorrect answers within 60 s.

### Outcomes

The primary endpoint was the change from baseline in matching-pair test scores and response times (i.e., the time to answer the matching-pair test) after 24 weeks (6 months) of nusinersen treatment. Additional endpoints were the change from baseline in: the number of correctly matched pairs at 6 months; CHOP-INTEND scores on items 1–7 after 24 weeks (6 months) of nusinersen; PedQL-NM scores, and NRS scores after 24 weeks of nusinersen treatment.

### Safety assessment

All potential AEs were recorded and reported to the sponsor (Biogen). AEs were assessed for their relationship to nusinersen and for their severity.

### Statistical analysis

As an exploratory analysis, no sample size was predefined. All data were analyzed descriptively using mean and standard deviation (SD) or median and interquartile range (IQR) for continuous variables, and count and percentage for categorical variables. Changes from baseline at Weeks 9 and 24 were evaluated by dividing the value of the parameter at that time point by the value at baseline. The Wilcoxon signed-rank test was used to calculate the *p*-value for the matching-pair test, CHOP-INTEND, and QoL scores.

## Results

### Patients

The study included seven patients with SMA type I with 2 or 3 copies of the *SMN2* gene. There were one male and six female patients, aged between 5 and 21 years (Table 1). All patients were non-ambulant and bedridden with an HFMSE score of 0 and CHOP-INTEND scores of between 0 and 11 at baseline. Six of the seven patients had a tracheostomy and gastrostomy. Most of them had severe scoliosis, three patients had strabismus, and two patients had nystagmus. In daily life, one patient was able to communicate with natural speech, four patients used a tablet-like communication device (Let's Chat; PHC holdings Corporation), and two patients communicated with eye movement due to limited motor function (Table 1).

**Table 1:** Patient characteristics.

	**Patient 1**	**Patient 2**	**Patient 3**	**Patient 4**	**Patient 5**	**Patient 6**	**Patient 7**
*SMN* gene (exon 7, 8) copy number							
*SMN1*	0, 0	0, 0	0, 1^a^	0, 0	0, 0	0, 0	0, 0
*SMN2*	2, 2	3, 3	2, 1	2, 2	2, 2	2, 2	2, 2
Age, years	10	17	7	11	21	14	5
Sex	F	F	F	M	F	F	F
Height, cm	131	140	120	125	151	123.5	102.6
Weight, kg	18.2	21.8	21	22.8	42	18.7	11.1
Patient's daily activity ability	Bedridden	Bedridden	Bedridden	Bedridden	Bedridden	Bedridden	Bedridden
Respiratory support	Tracheostomy, IPPV	NPPV	Tracheostomy, IPPV	Tracheostomy, IPPV	Tracheostomy, IPPV	Tracheostomy, IPPV	Tracheostomy, IPPV
Nutritional support	Gastrostoma	Oral ingestion	Gastrostoma	Gastrostoma	Gastrostoma	Gastrostoma	Gastrostoma
Strabismus present	No	No	Yes	Yes	No	Yes	No
Nystagmus present	Yes	No	No	Yes	No	No	No
Communication tool	Let's Chat^®^	Natural speech	Eye movement	Let's Chat^®^	Let's Chat^®^ Tobii, fine motor	Let's Chat^®^	Eye movement
Cobb angle	75	140	NA	60	NA	95	42
CHOP-INTEND score	6	11	0	0	0	1	4

a Patient 3 had one copy of exon 8 but no copies of exon 7 (where the stop codon is located), which indicates a deleted *SMN1* gene.

CHOP-INTEND, Children's Hospital of Philadelphia Infant Test of Neuromuscular Disorders; IPPV, invasive positive pressure ventilation; NA, not applicable; NPPV, non-invasive positive pressure ventilation; SMN, survival motor neuron.

### Matching-pair test and CHOP-INTEND results

Level 1 matching-pair tests were completed by all seven patients at each assessment, level 2 tests by five patients, and level 3 tests by three patients (Table 2). A planned statistical analysis was performed; however, some of the data could not be analyzed due to missing data values, so statistical analysis was conducted only on parameters that had values for ≥5 patients. Although there were no significant differences in the statistically analyzed data, the median change from baseline in the percentage of correct answers tended to increase at Week 9 (33.4%) for level 1, and at Week 9 (50.1%) and Week 24 (45.8%) for level 2, and the median finishing time tended to decrease at each test level over time (Table 3).

**Table 2:** Number of actions performed by each individual patient in the matching-pair test.

	**Patient 1^a^**	**Patient 2^b^**	**Patient 3**	**Patient 4**	**Patient 5**	**Patient 6**	**Patient 7**
**Level 1**							
Baseline	3	3	0	0	3	3	2
Week 9	3	3	0	3	3	2	3
Week 24	3	3	2	3	3	3	5
**Level 2**							
Baseline	6	6	–	–	6	2	1
Week 9	6	6	–	–	6	10	3
Week 24	6	6	–	–	6	5	4
**Level 3**			–	–			
Baseline	10	10	–	–	–	–	1
Week 9	10	10	–	–	5	5	6
Week 24	10	10	–	–	6	3	2

a Finishing times-level 1, 16 s at baseline vs. 15 s at Week 9 and 15 s at Week 24; level 2, 32, 29, and 31 s, respectively; level 3, 54, 48, and 51 s, respectively.

b Finishing times-level 1, 16 s at baseline vs. 15 s at Week 9 and 15 s at Week 24; level 2, 31, 30 and 29 s, respectively; level 3, 51, 50, and 53 s, respectively.

**Table 3:** Rate of change (%) from baseline in matching-pair test parameters at Week 9 and Week 24.

	***n***	**Week 9**		**Week 24**	
		**Median (IQR)**	***P*-value^a^**	**Median (IQR)**	***P*-value^a^**
**Level 1**					
No. of mistakes at 60 s	7	0 (0, 0)	1.000	0 (0, 3)	0.375
No. of correct answers at 60 s	7	0 (0, 33.3)	0.250	0 (0, 33.3)	0.375
Questions answered correctly at 60 s, %	5	+33.4 (0, 49.9)	–	0 (0, 49.9)	–
Finishing time, s	7	−1.0 (−159.0, 0)	0.281	−1.0 (−60.0, 0)	0.281
No. of actions at 60 s	7	+1 (0, 3)	0.212	+3 (1, 5)	0.062
**Level 2**					
No. of mistakes at 60 s	5	0 (0, 2)	–	0 (0, 1)	–
No. of correct answers at 60 s	5	+16.7 (16.7, 26.7)	–	+16.7 (0, 23.8)	–
Questions answered correctly at 60 s, %	4	+50.1 (20.0, 97.3)	–	+45.8 (10.0, 92.9)	–
Finishing time, s	5	−3.0 (−167.0, −1.0)	–	−60.0 (−159.0, −2.0)	–
No. of actions at 60 s	5	+3 (2, 3)	–	+3 (3, 7)	–
**Level 3**					
No. of mistakes at 60 s	3	0 (0, 4)	–	0 (0, 0)	–
No. of correct answers at 60 s	3	0 (0, 6.7)	–	0 (0, 9.1)	–
Questions answered correctly at 60 s, %	2	0 (0, 0)	–	0 (0, 0)	–
Finishing time, s	3	−6.0 (−60.0, −1.0)	–	−3.0 (−60.0, 2.0)	–
No. of actions at 60 s	3	+3 (3, 4)	–	+7 (4, 7)	–

The change from baseline (%) was calculated by dividing the percentage of correct answers at Week 9 or 24, minus the percentage of correct answers at baseline, by the percentage of correct answers at baseline, × 100. Patients with a baseline score of 0 were excluded. Statistical analysis is not available for parameters assessed in ≤ 5 patients.

a Wilcoxon signed-rank test.

IQR, interquartile range; No., number.

Overall, there was no significant difference in the median number of actions at 60 s between baseline and Week 9 (+1 action, *p* = 0.212), or baseline and Week 24 (+3 actions, *p* = 0.062) in level 1 matching-pair tests (Table 3). There was a tendency for the median number of actions at 60 s to increase in levels 2 and 3 between baseline and Week 9 (+3 actions and +3 actions, respectively) and baseline and Week 24 (+3 actions and +7 actions, respectively) (Table 3). For level 1 tests, there were no significant differences in the median number of mistakes, correct answers, or median finishing time between baseline and Week 9 (0 mistakes, *p* = 1.00; 0 correct answers, *p* = 0.250; −1 s, *p* = 0.281), or baseline and Week 24 (0 mistakes, *p* = 0.375; 0 correct answers, *p* = 0.375; −1 s, *p* = 0.281) matching-pair tests in the whole patient group (Table 3).

The median CHOP-INTEND score was 1.0 at baseline, and showed a non-significant increase by a median of 0 at Week 9 [95% confidence interval (CI) −0.3, 1.5; *p* = 0.500], and at Week 24 (95% CI −0.8, 2.8; *p* = 0.375) (data not shown).

### QoL scores

PedsQL-NM score and NRS scores for each patient are shown in Supplementary Table S1. There was a trend toward an improvement in PedsQL-NM total score between baseline and Week 24 (change in mean of 8.6 points), as well as an increase in the NMD domain subscore (change in mean of 11.6 points), but none of the changes from baseline reached statistical significance (Table 4).

**Table 4:** Quality of life measured using the Pediatric Quality of Life inventory for patients with neuromuscular disease (PedsQL-NM) and symptom severity measured using a numerical rating scale (NRS).

	**Mean (SD) score**		
	**Baseline**	**Week 9**	**Week 24**
PedsQL-NM score	*N* = 6	*N* = 6	*N* = 6
Total (25 items)	35.0 (20.3)	37.0 (18.4)	43.6 (23.1)
NMD subscore (17 items)	27.2 (19.3)	32.4 (14.8)	38.8 (20.7)
Communication subscore (3 items)	40.3 (45.5)	36.1 (42.7)	40.3 (43.0)
Family function subscore (5 items)	59.7 (22.4)	53.3 (18.9)	61.7 (21.1)
NRS score	*N* = 7	*N* = 6	*N* = 6
Total (15 items)	75.0 (0.0)	72.2 (21.0)	81.3 (5.4)
Motor system subscore (4 items)	20.0 (0.0)	19.5 (4.8)	21.8 (1.7)
Breathing subscore (3 items)	15.0 (0.0)	14.7 (2.4)	15.7 (1.2)
GI tract subscore (4 items)	20.0 (0.0)	19.5 (5.0)	21.3 (2.2)
Sleep subscore (2 items)	10.0 (0.0)	10.6 (0.9)^a^	10.7 (1.0)
Fatigue subscore (2 items)	10.0 (0.0)	11.6 (1.5)^a^	11.8 (1.7)

Improvement is indicated by an increase in the PedsQL-NM score and the NRS score.

a n = 5.

GI, gastrointestinal; NMD, neuromuscular disease; SD, standard deviation.

The total NRS score also improved by 6.3 points between baseline and Week 24, but the change was not statistically significant (Table 4). NRS scores showed no worsening of fatigue or sleep between baseline and both Weeks 9 and 24.

### Safety

There was no severe AEs observed during this study. There was one report of hypokalemia, possibly related to nusinersen. One patient reported lumbar puncture-related headache (twice) and back pain (three times).

## Discussion

We conducted an exploratory study to evaluate fine motor function in SMA type I patients with limited gross motor function and HFMSE score of 0 at baseline. The effect of nusinersen treatment was observed by matching-pair test using an eye-tracking system, and the results demonstrated no statistically significant difference between baseline and Week 24 in the primary endpoint. While it was difficult to detect any improvement by objective measures, caregivers commented that patients' facial expressions were more clear and their eye movements were better, and they could more easily communicate with patients after starting treatment with nusinersen. Potential reasons for the lack of statistical effect on the primary endpoint are the small number of patients included in the study, the task settings (which should perhaps have included repetitive tasks or time limits), and that patient eye movements varied more than expected.

In the matching-pair tests, some patients showed a ceiling effect in which all tests were accurately completed within 60 s, while others had difficulty in performing the level 2 or 3 test. The physician inferred that patients 3 and 4 could only undertake the level 1 test, so these patients did not perform level 2 tests at any time point. Patient 5 did not perform the level 3 test at baseline because the physician noted that ocular fixation was difficult for her in the level 2 test. Patient 6 tried to perform the level 3 test, but the physician decided that it was too difficult for the patient to continue and did not enter the score because she had already shown fatigue at level 2. Although patients 5 and 6 could not perform level 3 test at baseline, they could perform the level 3 test from Week 9.

In this study, we analyzed observed values without imputation of missing data. We focused on eye-movement, especially ocular fixation, and analyzed “number of actions” that combined the number of correct and incorrect actions as an additional post hoc analysis. Excluding the two patients with ceiling effects, additional analyses were performed using five patients' data, and missing scores (as described above) were imputed as 0. In order to analyze total eye movement, the total number of actions were calculated by combining scores from levels 1–3 at each time point. The total number of actions tended to increase at Week 9 and 24 from the baseline in level 1–3 tests for Patients 3, 4, and 7, and in level 1 and 2 tests for Patients 5 and 6 (Supplementary Figure S1). In the matching-pair test, the gazing time for selection of the panel was set to 1 s, so it was difficult for subjects to perform the test if they were unable to gaze at the panel for 1 s. As expected, one of the reasons for the potential increase in the “number of actions” was that the subjects were able to gaze at the panel for at least 1 s, suggesting that nusinersen treatment possibly improved ocular fixation. The median change in scores from baseline in 5 subjects tended to increase by 5 points at Week 9 (not significant, *p* = 0.223) and 4 points at Week 24 (*p* = 0.079). Although there was no statistically significant difference, the improvement in score appeared to be stabilized at Week 24. It is speculated that analysis of a larger sample size may be able to provide more accurate result.

Eye-tracking devices have been already widely used as communication tools and are very helpful in improving QoL for patients who are unable to communicate by voice or gesture (16). Patients with advanced SMA who received nusinersen treatment generally showed no significant changes in gross motor function score but their caregivers report slight improvement in other parameters, such as respiratory function (34–36). Therefore, QoL is also regarded as an important evaluation item and is measured using various tools in SMA (37). We also evaluated QoL scores (NRS/PedsQL-NM) in this study, but there was no significant difference during the observation period in this small cohort of patients with advanced SMA type I.

Weaver et al. analyzed the change from baseline in QoL scores using PedsQL-NM in 35 patients with SMA type I–III who were assessed 1–2 years (mean 1.8 years) after starting treatment with nusinersen (38). Although that study included more patients than our study, they also found no significant change from baseline in PedsQL-NM when they analyzed all subjects; however, there were significant improvements from baseline in communication on the PedsQL-NM scale and in emotional functioning on the PedsQL-family impact scale (38).

While subjective evaluation is important, it is not recommended as the primary measure of treatment evaluation in SMA (39), and identifying the most appropriate objective measure of assessment is an important limitation to overcome, particularly in severely affected patients (40). Ocular function is relatively preserved until the end in neuromuscular diseases, but ocular fixation instabilities are also reported in MND (41). Although there are no reports comparing the differences in eye movement between SMA and other diseases, ocular fixation is considered to be important when a gaze input device is used by patients, and it is expected that maintaining this function greatly affects a patient's QoL. In addition, a method using ocular fixation is possibly useful for evaluating fine motor function in patients with neuromuscular diseases.

The limitations of this study include the small patient number (attributable to the disease rarity), the wide age range of enrolled patients, the short observation period, and the fact that children tend to have mood swings, which may affect their test performance. In addition, patients may become accustomed to performing the matching-pair test, and this may possibly affect the results. A ceiling effect of the matching-pair test was shown in patients with preserved eye movement, so a more difficult test would be required in order to evaluate a range of patients including those with preserved eye movements. Also, prior to the start of the study, only routine examination (rather than a detailed evaluation) was performed to determine whether the oculomotor function of each patient was stable. Cognitive function may have also influenced the matching-pair test results, but this association could not be examined in the current study.

In this exploratory study, there were no statistically significant differences in the primary endpoint results, however the assessment of ocular fixation provided important findings that could possibly capture improvements in fine movements. To our knowledge, there have been no previous studies evaluating the use of eye-tracking systems to assess the effect of nusinersen treatment on ocular fixation, which may be a new method for the evaluation of fine motor function in patients with advanced SMA in the future.

## Data availability statement

The raw data supporting the conclusions of this article will be made available by the authors, without undue reservation.

## Ethics statement

The study was approved by the Institutional Review Boards of all participating hospitals, as well as the Ethics Committee of the Kurume University (reference 18103). Written informed consent to participate in this study was provided by the participants' legal guardian/next of kin.

## Author contributions

KY, YYa, and MC designed and developed the protocol of the study and wrote the manuscript. KY, YYa, TM, MM, OK, YB, SM, MS, and TF enrolled patients. KY, YYa, TM, OK, YB, and MS collected and analyzed the data. TM, FI, MM, OK, KO, YB, CY, SM, MS, TF, MC, and YYu reviewed and approved all drafts of the article. All authors contributed to the article and approved the submitted version.

## Funding

This study received funding from Biogen Japan Ltd (Tokyo, Japan) (Grant number: JPN-SPN-17-11270). This medical writing assistance was funded by Biogen.

## Conflict of interest

This study received funding from Biogen Japan Ltd (Tokyo, Japan). The funder had the following involvement with the study: design, analysis and interpretation, writing and the decision to submit this article for publication. All named authors have contributed significantly to the intellectual content of the manuscript, and have critically reviewed all drafts and approved the final version for submission. Author KY has received speaker honoraria from Biogen Japan, Novartis Japan, and Chugai Pharmaceutical for activities unrelated to the present work. Author OK has received honoraria from Biogen Japan and UCB Japan for activities unrelated to the present work. Authors SM and TF have attended a Biogen Japan advisory board unrelated to the present work. Author YYu has received honoraria from Shionogi, Novartis, Takeda, Daiichi Sankyo, Novel Pharma and Janssen for activities unrelated to the present work. Author MC is an employee of Biogen Japan Ltd. The remaining authors declare that the research was conducted in the absence of any commercial or financial relationships that could be construed as a potential conflict of interest.

## Publisher's note

All claims expressed in this article are solely those of the authors and do not necessarily represent those of their affiliated organizations, or those of the publisher, the editors and the reviewers. Any product that may be evaluated in this article, or claim that may be made by its manufacturer, is not guaranteed or endorsed by the publisher.
