# Supplementary material for: Exploratory evaluation of an eye-tracking system in patients with advanced spinal muscular atrophy type I receiving nusinersen
Source: Front Neurol. 2022 Sep 30;13:918255. doi: 10.3389/fneur.2022.918255 (PMC9563313; doi:10.3389/fneur.2022.918255)
Supplement: Supplementary file 1 [file Data_Sheet_1.pdf]

Supplementary Material

Supplementary Material

1 Supplementary Figures and Tables

1.1 Supplementary Figures

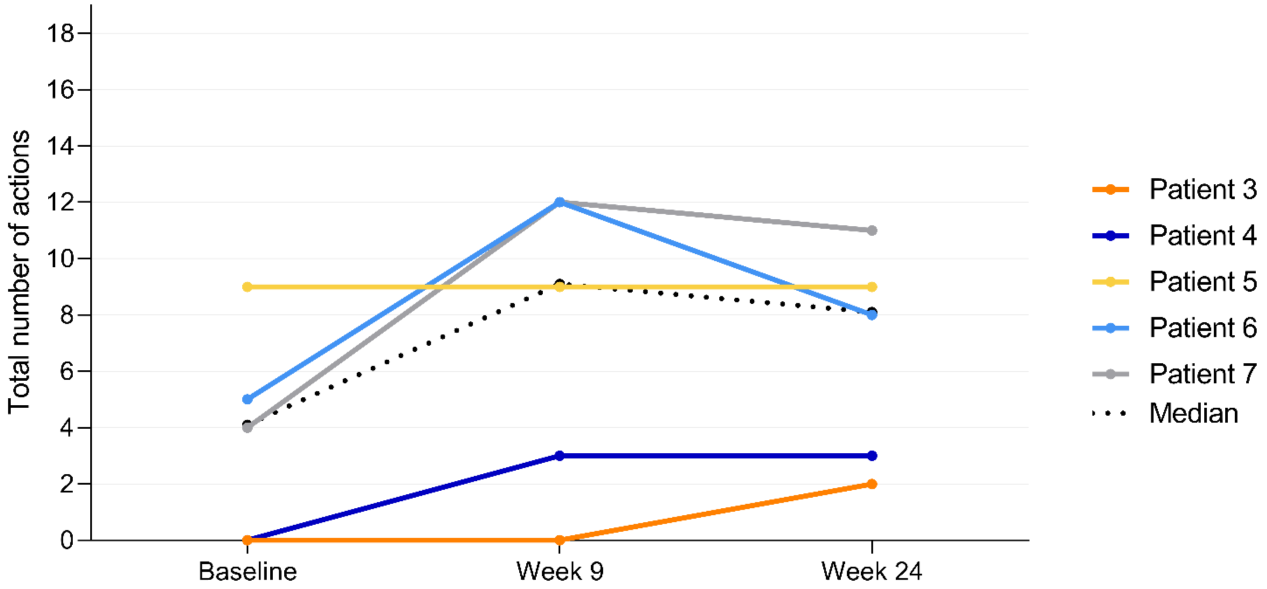

Supplementary Figure S1. Total number of actions at baseline, Week 9, and Week 24 in the cohort of patients excluding those who showed ceiling effects (Patients 1 and 2). Action count includes numbers of mistakes + number of correct answers. The median values at baseline, Week 9 and Week 24 are shown by the black dotted line. Analyses were performed using data from Patients 3–7. The level 3 test scores for Patients 5 and 6 are excluded.

1.2 Supplementary Tables

Supplementary Table S1. Individual CHOP-INTEND, PedsQL-NM and NRS scores at each assessment.

	Patient 1	Patient 2	Patient 3	Patient 4	Patient 5	Patient 6	Patient 7
**CHOP-INTEND**							
Baseline	10	11	0	0	0	1	4
Week 9	8	13	0	0	0	1	4
Week 24	5	16	0	1	2	1	4
**PedsQL-NM**							
Disease symptoms							
Baseline	34	47	0	19	49	–	15
Week 9	44	47	24	19	46	–	15
Week 24	59	65	18	29	46	–	16
Communication							
Baseline	75	100	0	0	67	–	0
Week 9	50	100	0	0	67	–	0
Week 24	67	100	0	8	67	–	0
Family function							
Baseline	65	65	83	35	80	–	30
Week 9	60	65	50	35	80	–	30
Week 24	70	90	45	45	80	–	40
**Total**							
Baseline	45	57	15	20	57	–	16
Week 9	48	57	26	20	55	–	16
Week 24	62	74	22	30	55	–	19
**NRS score**			–	–			
Motor function							
Baseline	20	20	20	20	20	20	20
Week 9	21	20	10	23	22	–	21
Week 24	23	20	20	23	24	–	21
Breathing							
Baseline	15	15	15	15	15	15	15
Week 9	15	15	10	17	15	–	16
Week 24	15	15	15	18	15	–	16
Gastrointestinal							
Baseline	20	20	20	20	20	20	–
Week 9	20	24	10	23	20	–	–
Week 24	20	25	20	23	20	–	–
Sleep							
Baseline	10	10	10	10	10	10	–
Week 9	10	12		11	10	–	–

Week 24	10	12	10	12	10	–	–
Fatigue							
Baseline	10	10	10	10	10	10	–
Week 9	11	14		12	11	–	–
Week 24	11	14	10	13	13	–	–
**Total**							
Baseline	75	75	75	75	75	75	1
Week 9	77	85	30	86	78	–	6
Week 24	79	86	75	89	82	–	2

CHOP-INTEND, Children's Hospital of Philadelphia Infant Test of Neuromuscular Disorders; NRS, numerical rating scale; PedsQL-NM, Pediatric Quality of Life inventory for patients with Neuromuscular Disease.
